# A Retrospective Review of Physician-related Patient Complaints from a Tertiary Pediatric Hospital

**DOI:** 10.1097/pq9.0000000000000136

**Published:** 2019-02-13

**Authors:** David Chaulk, Carsten Krueger, Antonia S. Stang

**Affiliations:** From the * Division of Pediatric Emergency Medicine, University of Utah, Salt Lake City, Utah; † The Hospital For Sick Children, University of Toronto, Toronto, Ontario, Canada; ‡ Departments of Pediatrics, Community Health Sciences and Emergency Medicine, University of Calgary, Calgary, Alberta, Canada.

## Abstract

**Background:**

Trends in patient concerns can identify systematic problems in health care delivery that may not be detected when addressing individual concerns. It can be difficult identifying trends without using a standardized taxonomy. The study objectives were to describe patient complaints from a tertiary care pediatric hospital and categorize them using a standardized complaint taxonomy.

**Methods:**

Physician-based patient complaints were compiled from April 2011 to May 2014 from a tertiary pediatric hospital. These complaints were coded independently by 2 reviewers using the Reader taxonomy, a published standardized taxonomy. Complaints were placed into 3 domains: clinical, management, and relationships then organized into categories. Inter-rater reliability for domain classification between the 2 reviewers was calculated using Cohen’s unweighted κ.

**Results:**

Eighty-seven patient complaints were identified, representing approximately 1 per 10,000 physician–patient encounters. Half (48/87) were related to care in the emergency department. When adjusted for volume, pediatric hospital medicine had the highest number of complaints, with 12.1 per 10,000 encounters. The majority of patient complaints, 66% (57/87), were of the clinical domain (κ = 0.61). Sixty percent (52/87) were in the relationship domain (κ = 0.68), and 16% (14/87) were in the management domain (κ = 0.65).

**Conclusions:**

We found a low overall complaint rate. Our results indicate that interventions to improve patient experience should initially be targeted at emergency and hospital medicine on the clinical and relationship domains. The inter-rater reliability of the Reader taxonomy was moderate with implications for processing patient complaints at a hospital level.

## INTRODUCTION

Analysis of patient complaints can help healthcare organizations detect trends in patient safety concerns informing the implementation of evidence-based improvement interventions.^1–5^ Similarly, trends in patient concerns can assist in the identification of systematic problems in health care delivery that may not be detected when addressing individual concerns.^6^ There are also associations between patient concerns and rates of malpractice suits.^5^

**Fig. 1.**
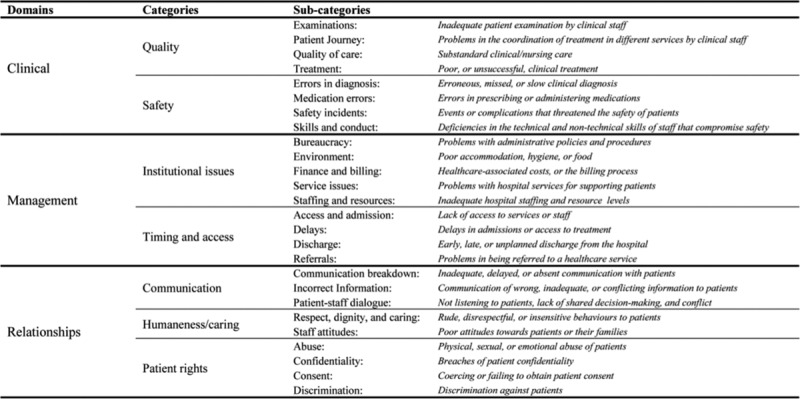
The patient complaint categorization taxonomy developed by Reader et al.^5^ Each complaint is assigned ≥1 domain, category, and subcategory as applicable. Reproduced from *BMJ Qual Saf*. 2014;23:678–689. Adaptations are themselves works protected by copyright. So in order to publish this adaptation, authorization must be obtained both from the owner of the copyright in the original work and from the owner of copyright in the translation or adaptation.

Healthcare organizations receive large volumes of patient concerns making it difficult to identify trends without the use of a standardized taxonomy. There are a number of these cited in the literature including Montini et al^4^ and Reader et al.^5^ In this analysis, authors chose the Reader taxonomy due to the size and rigor used in its development. Using a systematic review, the Reader taxonomy (Fig. 1) incorporated findings from 59 studies of patient complaints including almost 89,000 distinct complaints. The systematic review studied all patient concerns and did not focus on a particular demography, patient setting, or healthcare organization type. While there have been previous studies done regarding pediatric patient complaints, they were particular to specific care settings such as emergency departments.^7^ The lack of data on patient complaints from an entire children’s hospital represents a gap in the current literature. Data on hospital-wide complaints would enable comparison across settings, and identification of areas where intervention may be needed to improve the patient experience. The objectives of this project were to (1) describe patient complaints at a tertiary care children’s hospital; (2) characterize patient complaints using a previously published taxonomy; and (3) measure the inter-rater reliability of categorization using the taxonomy.

## METHODS

This study took place at the Alberta Children’s Hospital (ACH), which is a tertiary-care pediatric hospital in Calgary, Alberta, Canada. The ACH, a level 1 trauma center, is a referral center for southern Alberta, southwestern Saskatchewan, and southeastern British Columbia with approximately 140 inpatient beds and >70,000 emergency department visits per year. All major pediatric medical and surgical subspecialties are represented.

From April 1, 2011, to May 30, 2014, an author (D.C.) collected all ACH physician-related complaints received by the office of patient concerns. D.C. received other physician-related complaints through various administrative channels, and some directly from patients. The office of patient concerns received the majority of the patient concerns. All physician-related patient concerns received in the study period were included. The Conjoint Health Research Ethics Board of the University of Calgary provided ethics approval.

RedCap (version: 6.12.0; Vanderbilt University, Nashville, Tenn.), a web-based application designed to support data capture for research studies, stored clinical data about these complaints.^8^ Coding of patient complaints was according to the physician group or groups involved (anesthesia, mental health, pediatric emergency medicine, pediatric hospital medicine, pediatric subspecialties, pediatric general surgery, pediatric surgical subspecialties, and radiology). All deidentified patient complaints were then independently coded by 2 reviewers (D.C., C.K.) according to the Reader taxonomy^5^ (Fig. 1). The patient complaint rate is calculated per 10,000 patient encounters. The overall number of encounters is based on both inpatient and ambulatory data. The number of patient encounters is obtained from hospital administrative data. For inpatient data, it is based on the number of admissions to that particular service and not the absolute number of patient contacts during that admission. For ambulatory data, this number includes each individual encounter. Although the number of patient visits does not include inpatient consults, the complaint may come from a consult where the patient is admitted to a different service.

The Reader taxonomy divided patient complaints into 3 major domains: clinical, management, and relationships. Clinical includes the categories quality and safety. The clinical domain pertains to patient reports on poor quality care and safety incidents.^5^ Management includes the categories institutional issues, and timing and access and pertains to problems in waiting times/access to care and institutional management.^5^ The relationship domain includes the categories communication, humaneness/caring, and patient rights. It considers patient complaints on interactions and experiences of healthcare professionals.^5^ Reader’s taxonomy separated categories into subcategories. Each complaint could fall under multiple domains, and similarly in multiple categories.

Hospital administrative data (on the volume of inpatient and ambulatory visits overall and by physician group) were obtained through the Child Health Annual Report for the years 2011–2014. Descriptive statistics (means, medians, and proportions) were used to describe the volume of patient visits overall and by physician group (ie, complaints per physician group and complaints per patient contact). A similar approach was employed to describe the proportion of complaints that fit into each domain and category of the Reader taxonomy, overall and by physician group. Inter-rater reliability between the 2 independent reviewers was calculated using Cohen’s unweighted κ.^9^ A κ of 1 indicates perfect inter-rater agreement, whereas a κ of 0 indicates no more agreement than chance.^9^

## RESULTS

The analysis identified a total of 87 distinct patient complaints giving a rate of approximately 1 complaint per 10,000 patient encounters. Pediatric emergency medicine physicians accounted for 48 (47.1%) complaints, which represented 2.3 complaints per 10,000 patient encounters. Table 1 lists the percentage of complaints directed toward other physician groups. Pediatric subspecialty includes all pediatric subspecialty medical physicians that provide care at ACH. Pediatric surgical subspecialties include all pediatric surgical subspecialties including orthopedics.

**Table 1.**
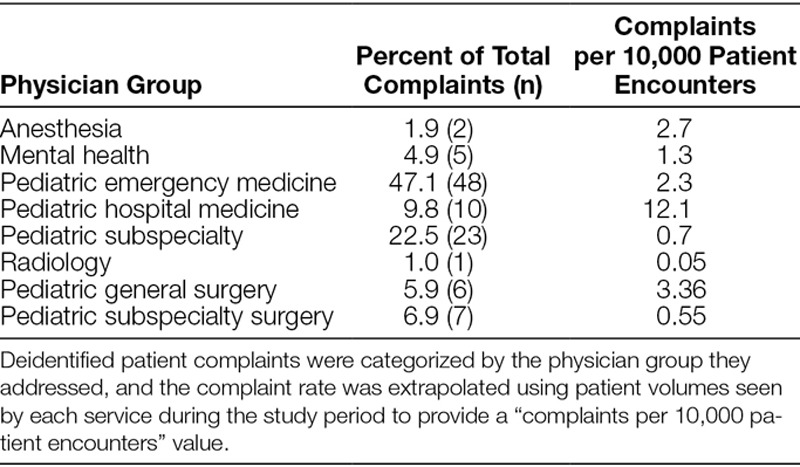
Distribution of Patient Complaints by Physician Group

Table 2 lists the results for domain and category classification. Recall that in our classification of complaints, domains and categories are not mutually exclusive, and therefore, multiple domains and categories can be assigned to the same complaint as appropriate.

**Table 2.**
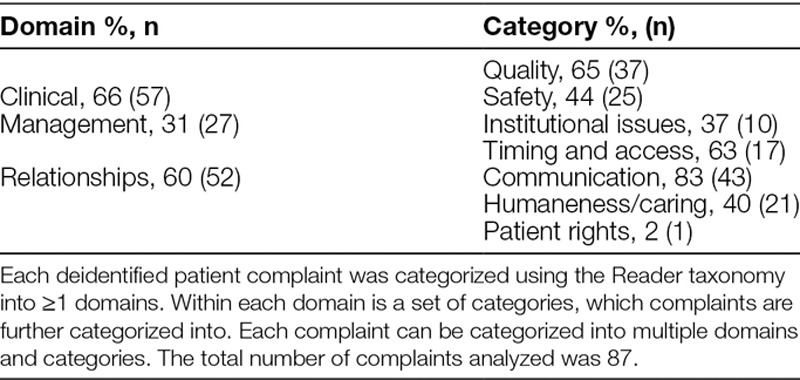
Distribution of Patient Complaints by Domain and Category

The majority (66%) of patient complaints are clinical. Thirty-one percentage of complaints are categorized as management, of which 63% are related to delays in accessing care. Over half of physician-based complaints are in the relationship domain (60%). Of these, 83% are related to poor communication.

The inter-rater reliability was found to be moderate across each of the 3 domain classifications. In particular, Cohen’s unweighted κ for the clinical domain was 0.61 (95% CI, 0.44–0.78), for the management domain was 0.65 (95%, CI 0.48–0.82), and for the relationship domain was 0.68 (95% CI, 0.53–0.83).

## DISCUSSION

Overall, there were few patient complaints, but several trends identified. Analysis by provider showed that the highest number of complaints was related to care provided by pediatric emergency department physicians. This result is not unexpected. Previous research has identified emergency medicine as particularly high risk for patient safety events, with the most common events related to management and diagnosis.^10,11^ The high acuity, volume, short period of interaction, and episodic nature of emergency medicine lead to lack of diagnostic differentiation, and potential communication failures. Our rate of 2.3 complaints per 10,000 emergency department encounters is much lower than previously reported in the literature for children; 1 study by Taylor et al^7^ in Australia reported a rate of 0.9 complaints per thousand emergency department encounters for patients 1–20 years old. This difference may be due to the robust nature of Australia’s Health Complaint Information Program compared with our infrastructure but is most likely because the complaints reviewed in Taylor et al^7^ were not physician-specific. When encounter volume is considered, the highest proportion of complaints per 10,000 patient encounters was related to pediatric hospitalist care, with a rate of 12.1 per 10,000. Similarly, this result is not unexpected. When patients are admitted to a pediatric hospital medicine service, there is typically less diagnostic clarity when compared with a pediatric subspecialty or surgical service. Overall, the majority of complaints were related to the clinical domain and specifically to the quality of care. There was subjectivity in categorizing the complaints beyond the 3 domains, and as such, many complaints are classified into multiple categories.

Previous work on complaints in the ED literature has linked patient complaints to patient safety events.^12^ Similarly, research in a pediatric hospital found that a family-based system for reporting adverse events helped identify patient safety concerns that were not identified by other reporting mechanisms.^13^ Although our study was not designed to link patient complaints to patient safety events, our results in the context of existing literature highlight the importance of soliciting patient complaints and suggest that categorizing and addressing them in a systematic manner has the potential to improve the quality and safety of care provided.

Ours is not the first study to apply the Reader taxonomy to a set of patient complaints; Harrison et al^14^ have conducted a study where 138 serious complaints are drawn from 67 independent hospitals in New South Wales, and, with a sample of 14 complaints, determined the inter-rater reliability to be high (k = 0.92). Our study differs in that Harrison et al^14^ were limited to serious complaints and found that the events related to the complaint had a mortality rate of 38%, whereas the current study included any physician-related patient complaint regardless of severity. Our study found higher rates of complaints involving an aspect of communication and interpersonal relationships, which is consistent with the findings of previous patient complaint analyses in Singapore and Sweden.^15,16^

One concern Harrison et al^14^ advanced was that classifying complaints into >1 domain and/or category could reduce the clarity of the information gathered. In the present study, we show that even with a larger sample size and a more diverse pool of physician-related patient complaints that the inter-rater reliability of the Reader taxonomy is still moderate. Rather than compromising the quality of data derived from patient complaints, we conclude that allowing complaints to fall under multiple domains and categories will acknowledge their nuanced nature, perhaps providing a more reliable estimate of the incidence of each type of complaint.

Our results make an important contribution to the existing literature by applying the Reader taxonomy to physician-related patient complaints drawn from an entire pediatric hospital, and by assessing the inter-rater reliability of the Reader taxonomy. Use of the Reader taxonomy in a pediatric hospital may serve as a valuable way of classifying patient complaints, which would provide evidence to support the need for changes in hospital policies and procedures. The ability to systematically track and categorize complaints will enable clinicians and administrators to identify the specific clinical areas, and aspects of patient care, which require intervention to improve the patient experience. Knowledge of the specific domains that complaints fall in can be used to design specific interventions that are targeted to the clinical context. Over half of the physician-based complaints in our sample are in the relationship domain, the majority of those related to communication. Interventions that have shown to be effective in improving doctor–patient communication include communication training for physicians, encounter facilitation tools, and decision aids.^17,18^

As with previous studies, our study faces a few limitations intrinsic to research evaluating patient complaints. It is probable that some patient complaints are addressed in “hallway talks” and never come through traditional administrative channels. As a result, the volume and nature of these complaints as well as their impact on the category proportions are unknown. It is possible that our patient complaint rate is skewed. As an example, it is possible that a patient was admitted to 1 service with another service consulting. If a complaint is received about the consulting service, it will not be reflected in the denominator. Furthermore, patient complaints come in multiple forms and include a varying amount of detail. These factors could limit the utility of a patient complaint taxonomy in a healthcare setting unless a standardized complaint form is created to aid classification into the relevant domains and categories. Finally, differences in payer mix and accessibility in healthcare systems outside Canada may limit the external validity of our study.

## CONCLUSIONS

Patient complaints are a valuable source of information that can be used to improve the care future patients receive, and trends in complaints can identify potential areas for quality improvement. Specialized taxonomies for categorizing complaints allow for better tracking of complaint frequency and subject matter over time. Our results suggest that patient complaints are relatively rare, that they most frequently involve clinical care and interpersonal relationships, and that the inter-rater reliability of the Reader taxonomy is acceptable. Heath care organizations do not typically utilize standardized taxonomies when addressing patient complaints, and our results suggest that the Reader taxonomy could be used to categorize complaints reliably. Categorizing patient complaints may help identify institutional issues that would remain undiscovered when complaints are reviewed independently. Our results from a tertiary care children’s hospital indicate that interventions to improve patient experience should initially be targeted at emergency and hospital medicine and should focus on the clinical and relationship domains. Future study should focus on the prospective use of the Reader taxonomy in categorizing patient complaints, and the potential link between patient complaints and patient safety events, across multiple pediatric centers and/or health systems.

## DISCLOSURE

The authors have no financial interest to declare in relation to the content of this article.
